# Immediate Implants in Posterior Extraction Sites: A Case Series Applying the Dual-Zone Therapeutic Concept With a Three-Year Follow-Up

**DOI:** 10.7759/cureus.54890

**Published:** 2024-02-25

**Authors:** Ahmed Bahaa, Abdallah M Bahaa, Nada El-Bagoury, Nora Khaled, Ahmed M Ibrahim

**Affiliations:** 1 Oral and Maxillofacial Surgery, Faculty of Dental Medicine, Al-Azhar University, Cairo, EGY; 2 Operations, Innovinity Medical Hub, Cairo, EGY; 3 Orthodontics, Faculty of Dental Medicine, Al-Azhar University, Cairo, EGY; 4 Orthodontics, Faculty of Dentistry, Misr International University, Cairo, EGY; 5 Orthodontics, Faculty of Dentistry, Ain Shams University, Cairo, EGY; 6 Research and Development, Innovinity Medical Hub, Cairo, EGY; 7 Endodontics, Faculty of Dentistry, Cairo University, Cairo, EGY

**Keywords:** atraumatic extraction, peri-implant marginal tissue health, marginal bone loss, immediate implant, dual-zone therapeutic concept

## Abstract

The study aims to present 11 immediately placed implants in posterior extraction sockets applying the dual-zone therapeutic concept. Five patients with non-restorable molars or premolars were treated with single or multiple immediate implants after atraumatic tooth extraction using a piezotome. The dual-zone therapeutic concept included grafting the jumping gap adjacent to the implant up to the gingival margin with a bovine xenograft. A screw-retained customized healing abutment was used to allow healing, and the implant loading was delayed for four to six months. All the patients were followed up for three years. Surgical complications, implant or prosthesis loss, and peri-implant marginal tissue health were assessed annually. No surgical complications or implant loss were observed during the follow-up visits. Peri-implant marginal tissue health showed excellent results with minimal marginal bone loss. Bone gain was evident in some cases. Using the dual-zone therapeutic concept with immediate implant placement in posterior extraction sockets showed promising results over three years.

## Introduction

Immediate implant placement after tooth extraction offers many advantages, such as shorter treatment time and fewer surgical procedures, which can lead to greater patient satisfaction. Furthermore, studies have shown that the survival and success rates of immediate implants are similar to those of those placed in healed edentulous ridges. The survival rates for immediate implant placement are as reasonable as, if not slightly better than, delayed implant placement. Recent research has found that implants installed immediately after tooth extraction have a survival rate of over 98% after a minimum of one year of follow-up, with slightly more marginal bone loss of 0.31 mm than delayed placement [1]. These results are on par with implant placement in healed sites, demonstrating five-year survival rates of up to 95% [1,2].

This implant placement protocol is particularly advantageous when placing a single or multiple adjacent implants. Preserving soft and hard tissues for aesthetic purposes is crucial, as modern patients have high expectations for the appearance of their replaced or repaired teeth. Additionally, this technique may prove beneficial in replacing failing posterior teeth and restoring proper chewing [3]. The most critical surgical step entails atraumatic tooth extraction without flap elevation. This is especially pertinent in the esthetic zone, which features the thinnest dimensions of the buccal bone plate and soft tissues in the buccal-palatal dimension [4]. The underlying rationale for this procedure is to preserve the remaining blood supply from both the periosteum and endosteum, thereby maximizing the healing potential [3].

Chu and colleagues introduced the dual-zone therapeutic concept (DZT) in managing immediate implant placement in 2012 to enhance esthetics in the anterior region and preserve the hard and soft tissue after extraction and implant placement [5]. The dual zone refers to the bone and the tissue zone apical and coronal to the immediately placed implant head, which could recede or collapse after tooth extraction. DZT consists of packing the bone graft material in the bone gap between the implant and the buccal plate until the free gingival margin. This is followed by prosthetic socket sealing using either a custom healing abutment or a provisional restoration [3,5]. DZT technique was researched mainly for immediate implant placement in the anterior area. However, few studies have reported it, and to our knowledge, no studies have used the same technique in posterior teeth [5-8]. This study presents a case series of 11 immediate implants placed in posterior extraction sockets with three years of follow-up using the DZT technique.

## Case presentation

Five patients with unrestorable single or multiple posterior teeth underwent the DZT technique with immediate implant placement after tooth extraction. These patients were treated at our private dental center, Innovinity Medical Hub, located in Heliopolis, Cairo, Egypt, between 2020 and 2023. All patients were non-smokers, in good health, and with good oral hygiene. Chief complaints included functional or masticatory problems caused by unrestorable posterior teeth, except for one case where second primary lower molars were retained. The mean age of the patients was 46.4 ± 17.4 years, with four females and one male. Among the five patients, two cases had two implants each for a three-unit bridge, with one having an additional single implant. One case had four single implants placed, and the remaining two instances each had one single implant placed. Tooth extraction was performed using a piezotome for atraumatic extraction.

After surgical debridement of the extraction sockets, a suitable diameter (ranging from 3.5 to 5.5 mm) root form implant with aggressive threads (RooTT®, United Kingdom) was placed in the socket 3 mm apically to the free gingival margin in a palatal or lingual position. No flaps were required during the tooth extraction or the implant placement, and the buccal bone walls were intact. A primary implant stability of 40 Ncm was achieved to allow immediate placement of a screw-retained customized healing abutment using flowable composite and rubber index before the grafting procedure. The custom healing abutment was replaced with a flat healing abutment. The jumping gap was packed with bovine xenograft bone of 0.5 to 1 mm particle size (Cerabone®, botiss, Germany) using a sterile amalgam carrier and a small narrow curved bone plugger to condense the graft material until the gap was filled to the free gingival margin. The graft material is set for 10 minutes until the blood clot is formed. The healing abutment is replaced by a custom screw-retained healing abutment, which seals and matures the graft material for 4 to 6 months before the first disconnection and impression making. The patient was advised not to brush the area for three days. A follow-up examination was carried out a week later, and proper oral hygiene instructions were given to all patients.

After disconnecting the custom healing abutment, an intraoral digital scanner was used to obtain a tissue-level digital impression of the implant sites. A digital abutment (titanium-based) was fabricated accordingly. The definitive restoration used was screw-retained zirconium crowns or bridges. A detailed step-by-step photograph of two cases with immediate implants placed using the DZT technique is shown in Figures 1-19.

**Figure 1:**
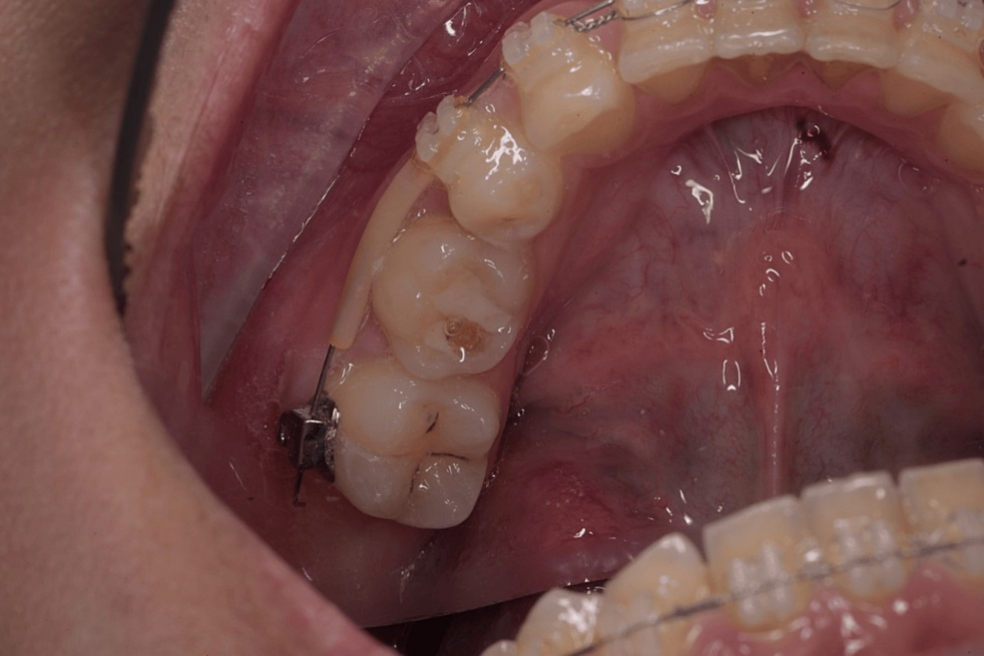
Retained mandibular 2nd molar to be replaced by an immediate implant.

**Figure 2:**
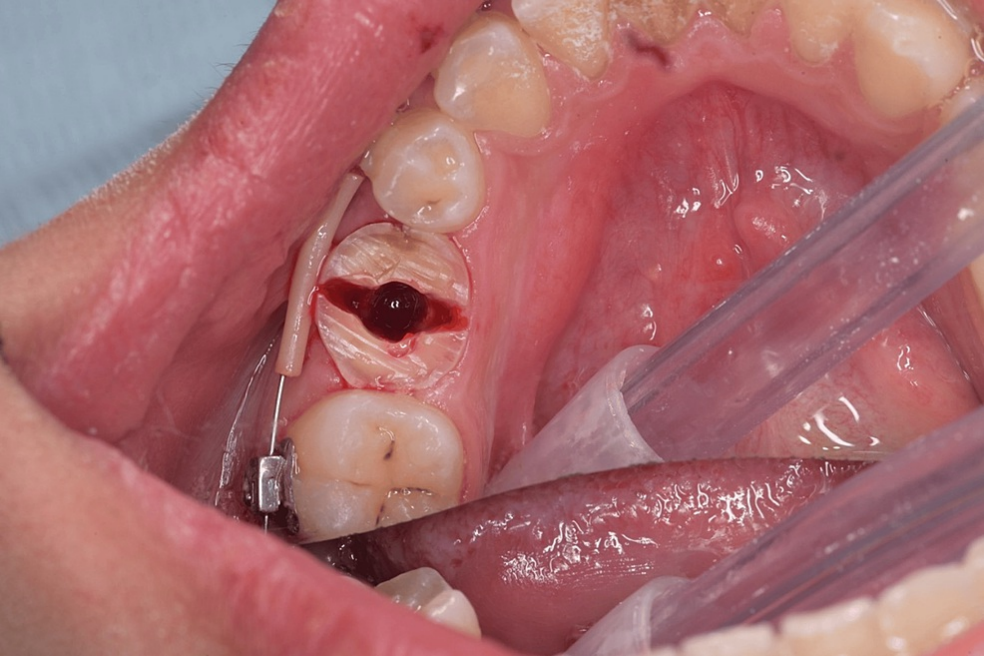
Sectioning of the tooth in a buccolingual direction for atraumatic extraction after crown reduction.

**Figure 3:**
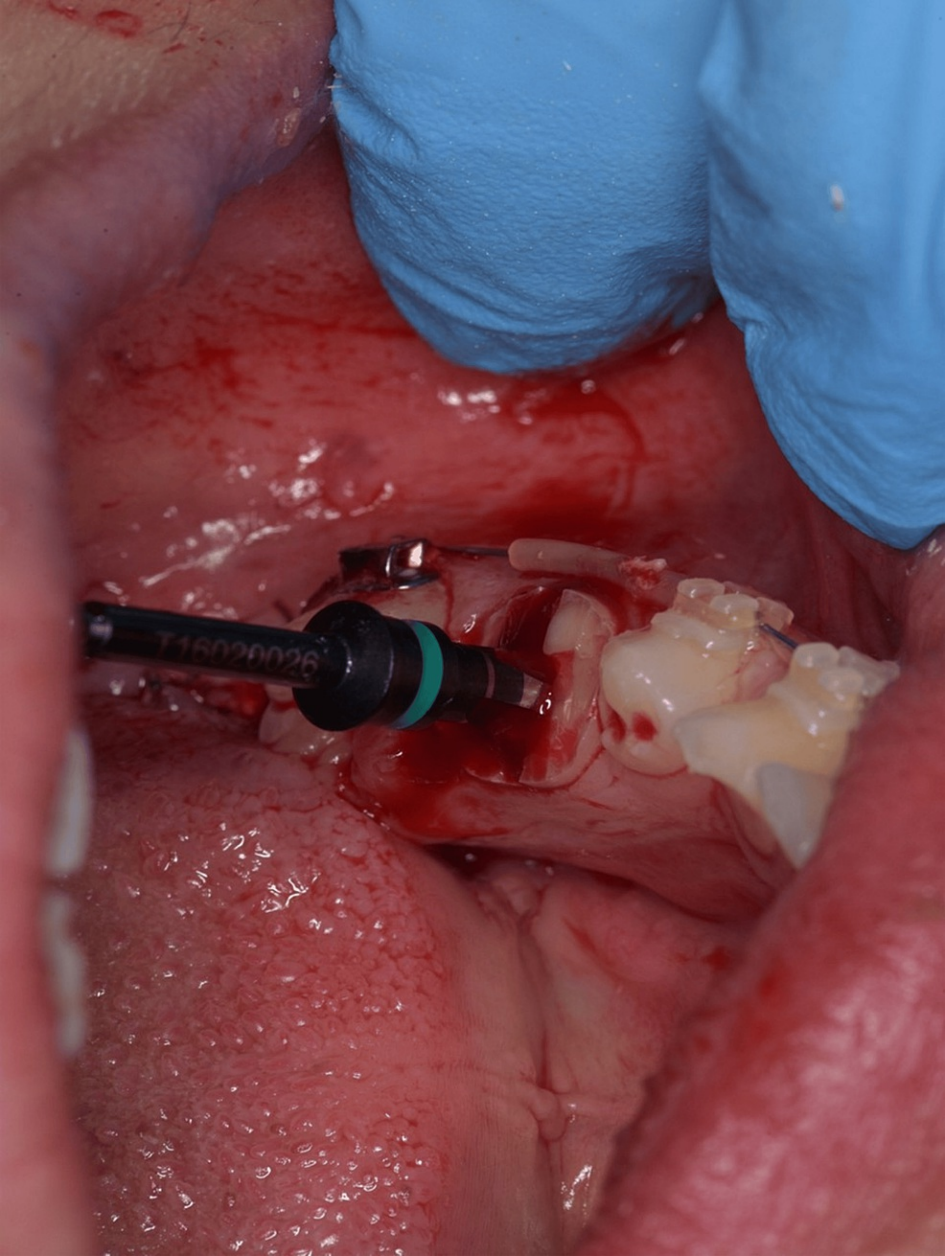
Implant drill in place after drilling.

**Figure 4:**
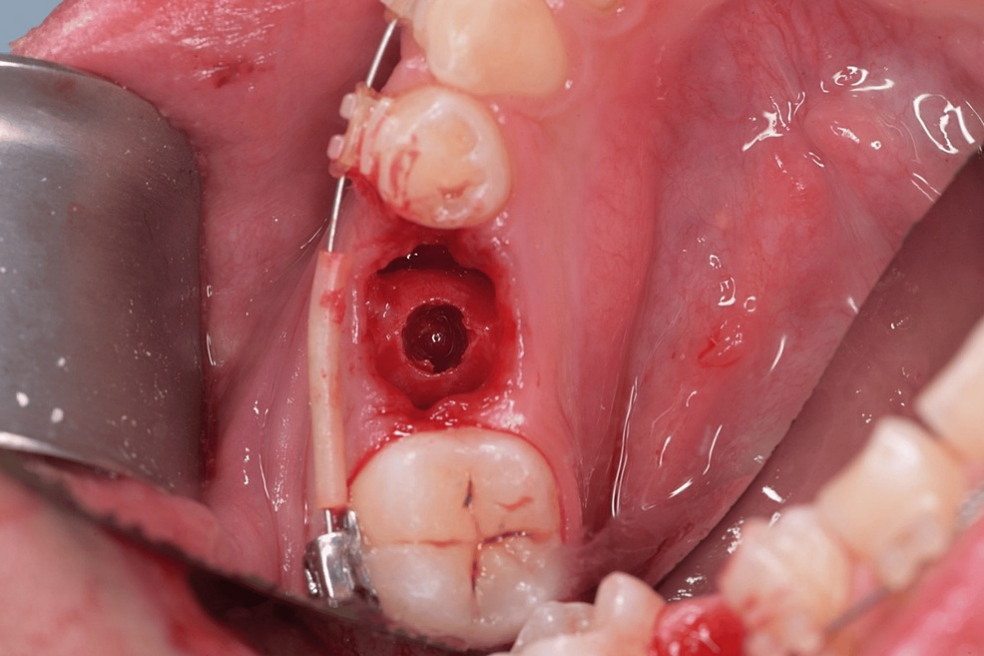
Prepared osteotomy site after implant drilling.

**Figure 5:**
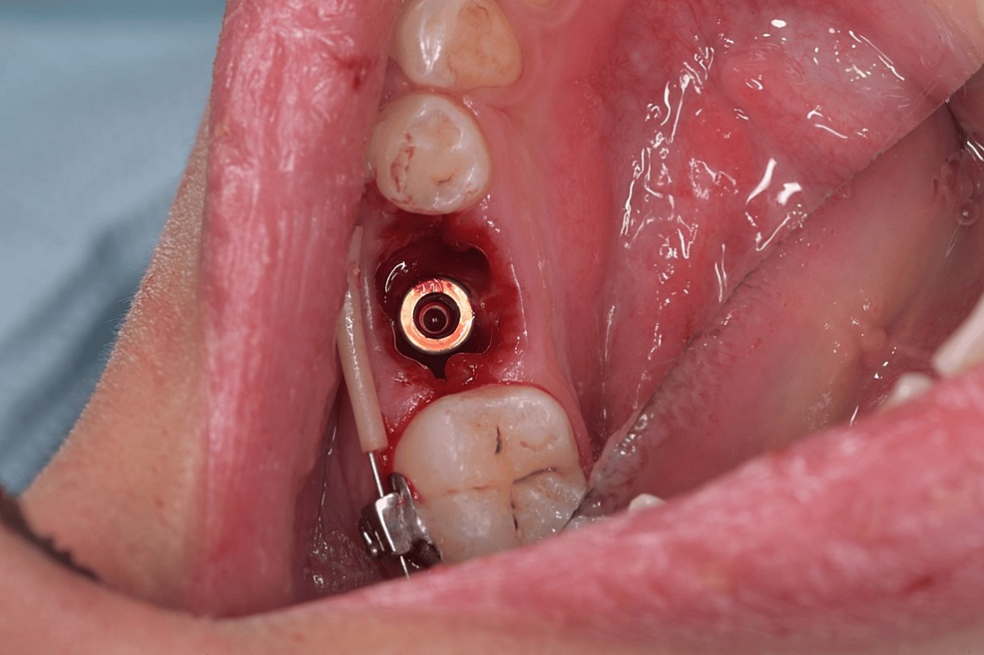
Root form implant in place.

**Figure 6:**
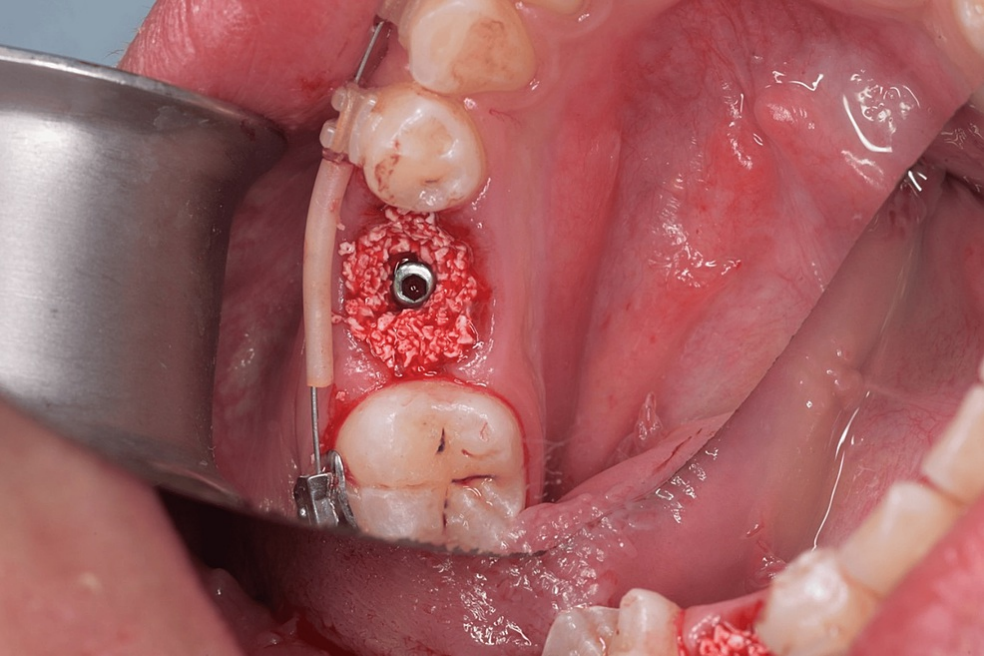
Bone graft filling the jumping gap until the free gingival margin.

**Figure 7:**
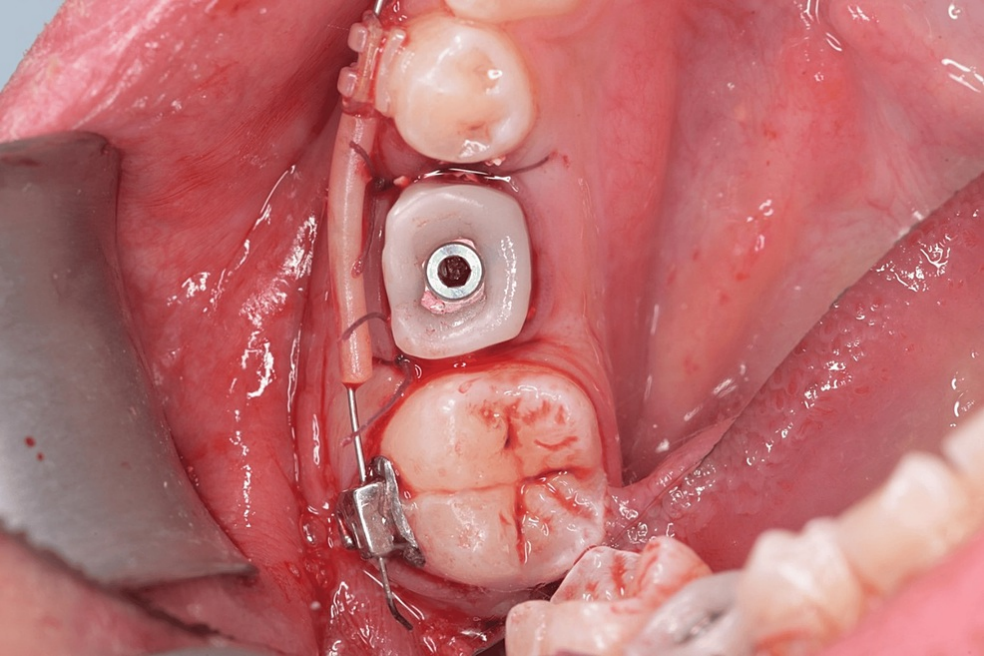
Screw-retained customized healing abutment in place.

**Figure 8:**
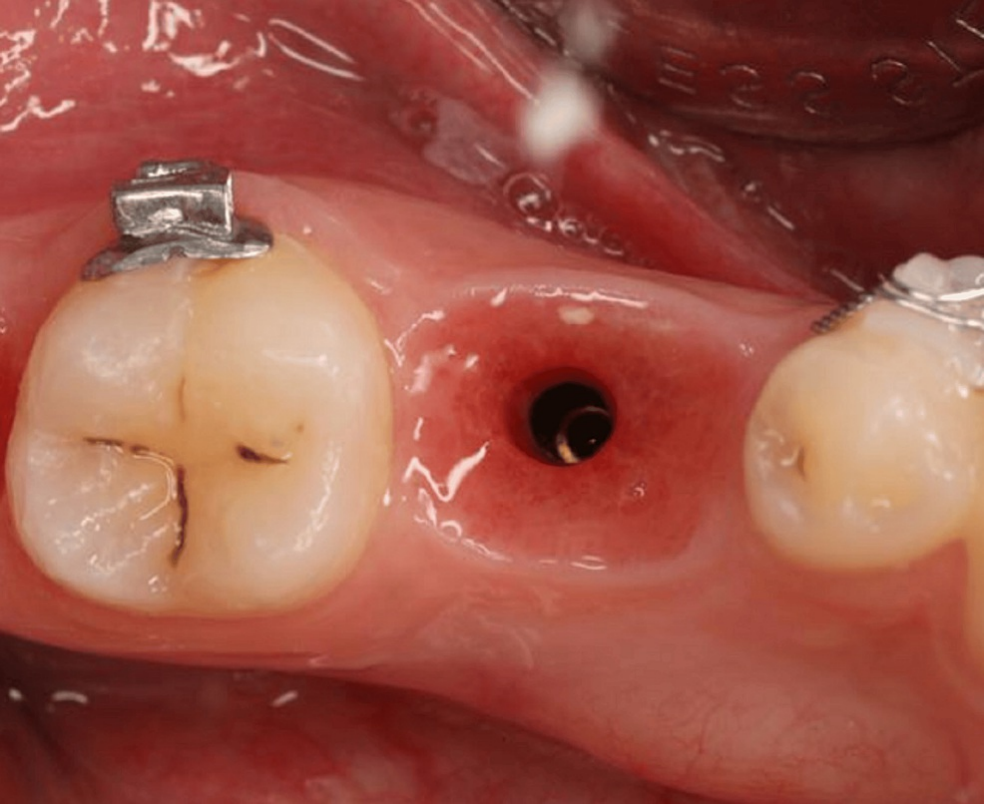
First disconnection after four to six months of healing.

**Figure 9:**
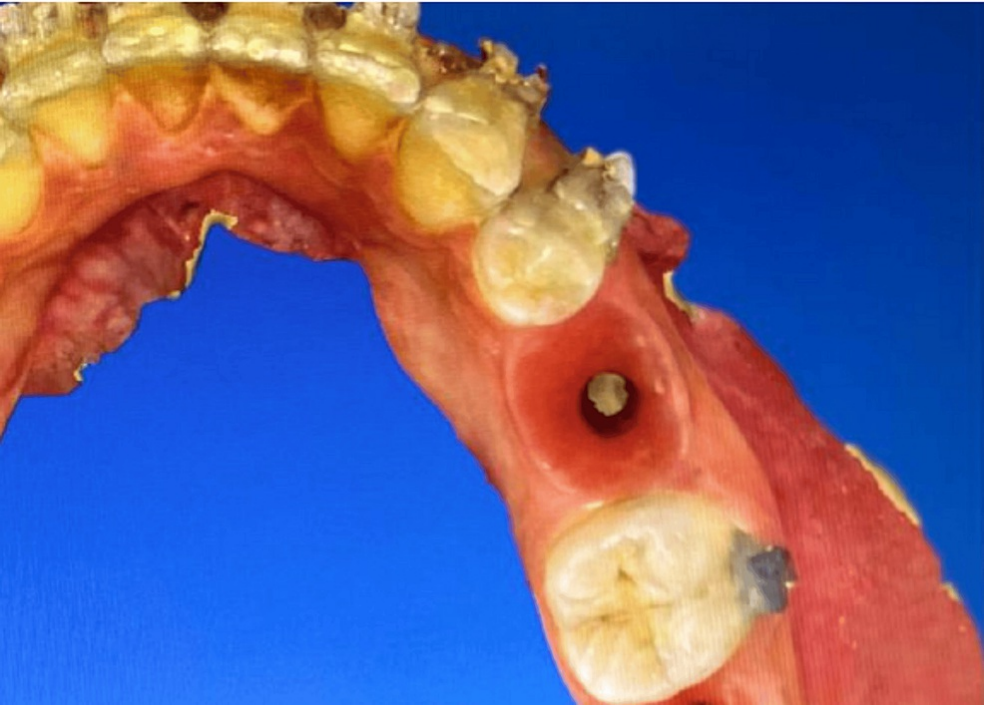
Digital impression using the digital intra-oral scanner.

**Figure 10:**
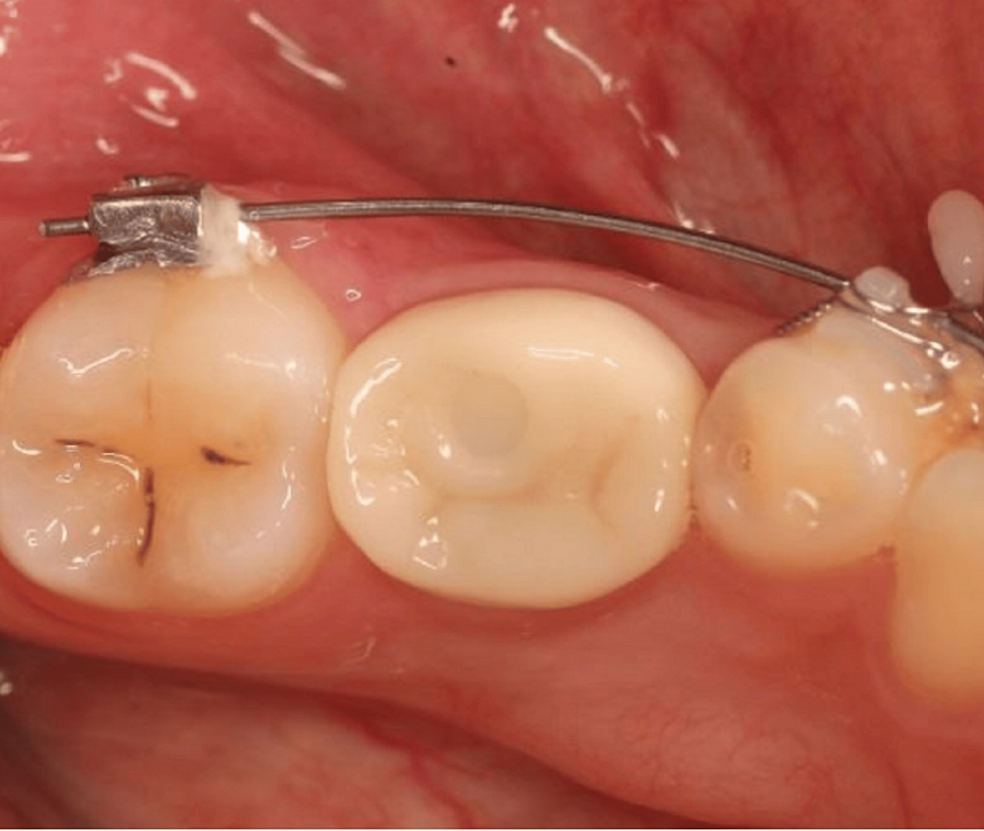
Definitive restoration (screw-retained zirconium crown).

**Figure 11:**
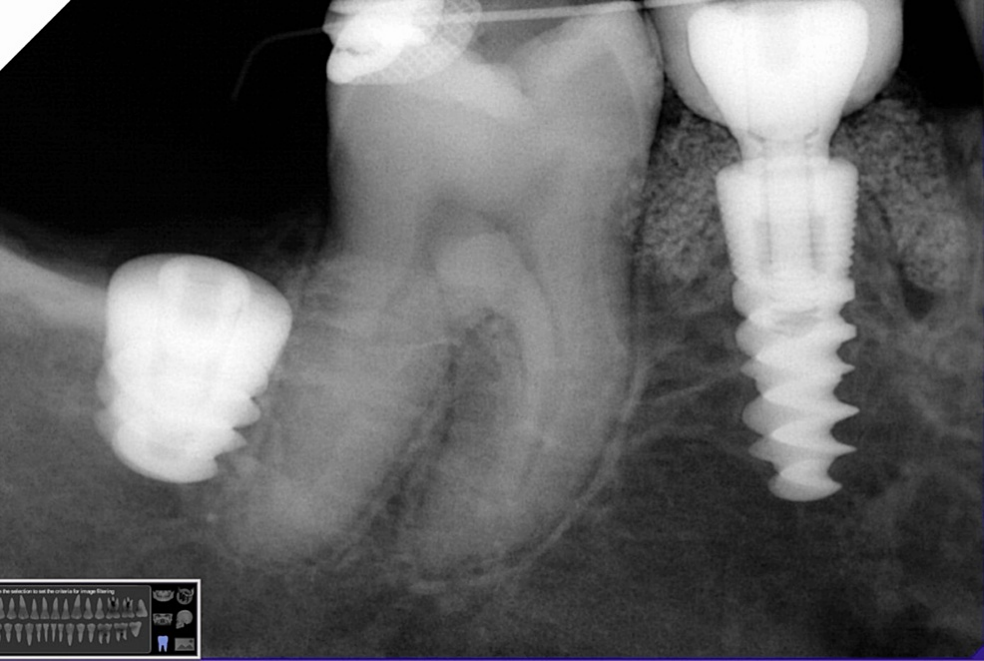
Periapical X-ray showing the implant with the definitive restoration.

**Figure 12:**
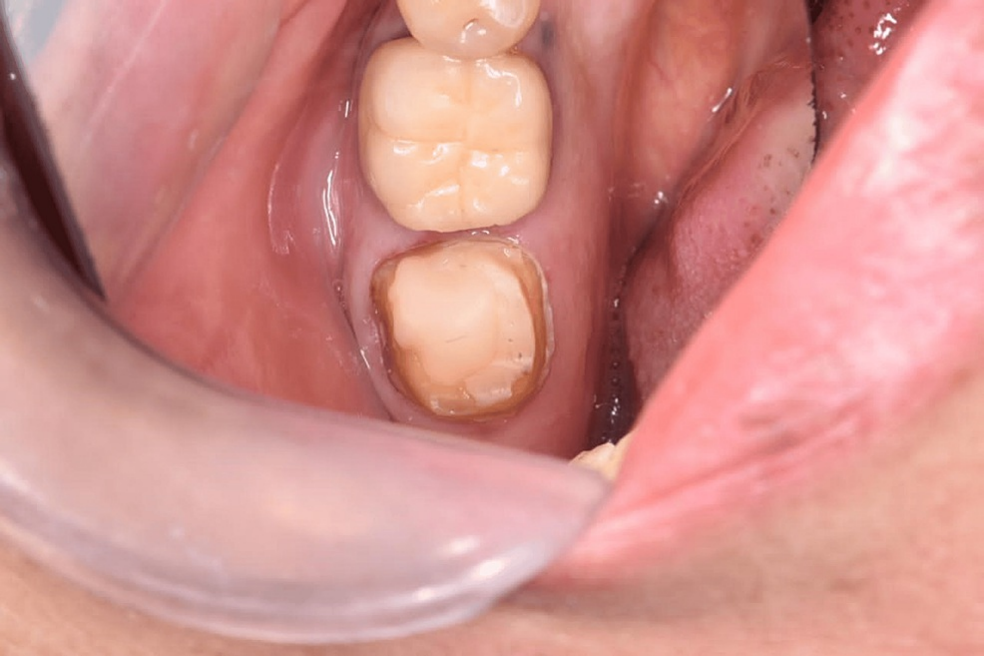
Non-restorable lower second mandibular molar for immediate implant replacement.

**Figure 13:**
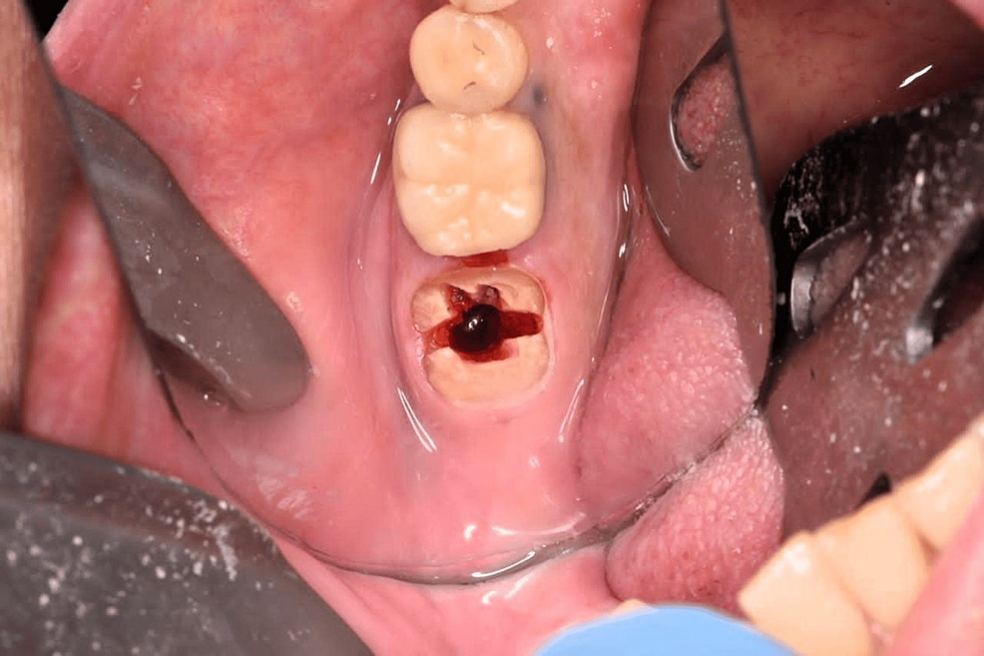
Sectioning of the tooth in a buccolingual direction for atraumatic extraction after crown reduction.

**Figure 14:**
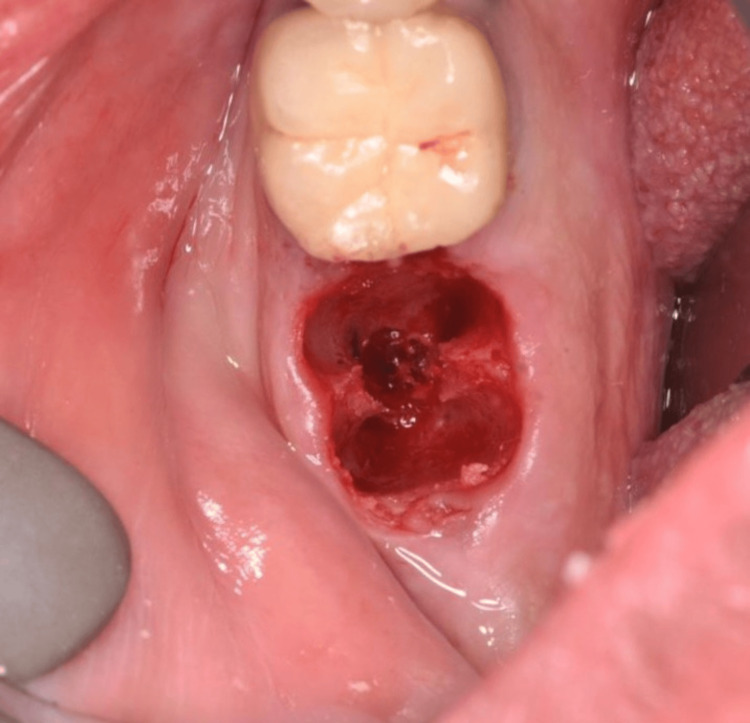
The prepared implant site after extraction using a piezotome.

**Figure 15:**
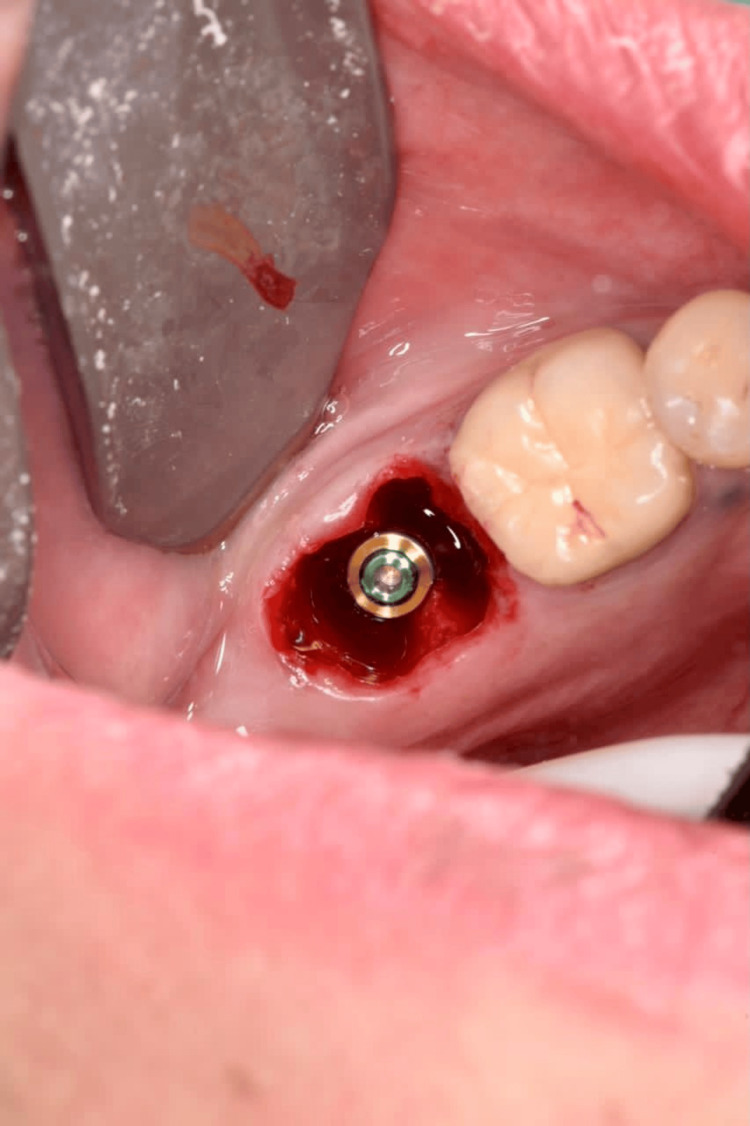
Root form implant in place.

**Figure 16:**
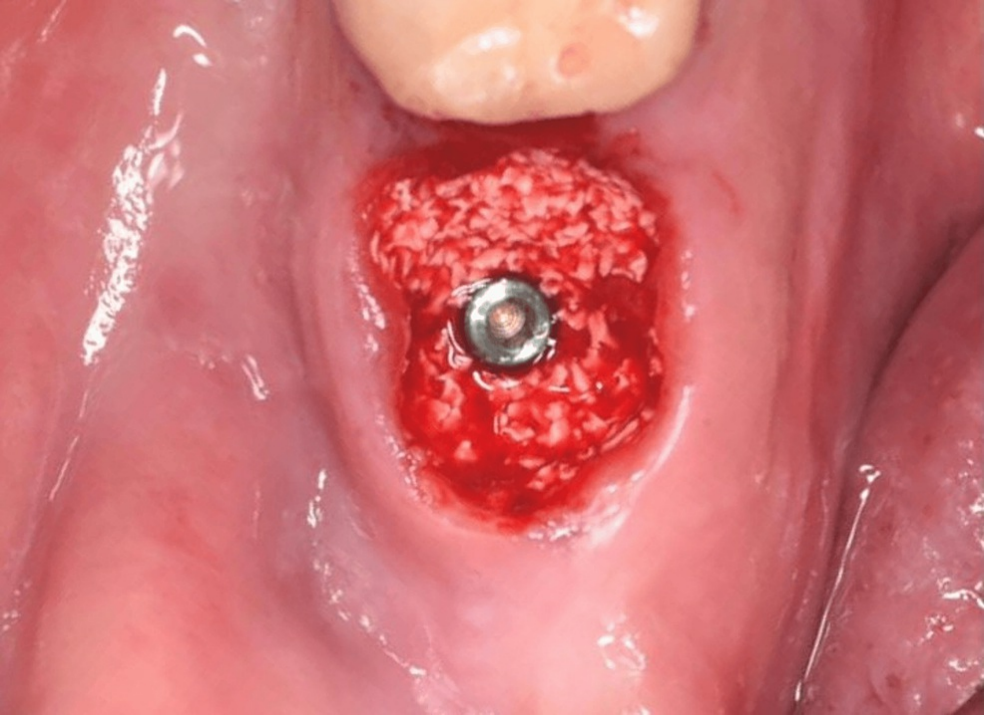
Bone graft filling the jumping gap until the free gingival margin.

**Figure 17:**
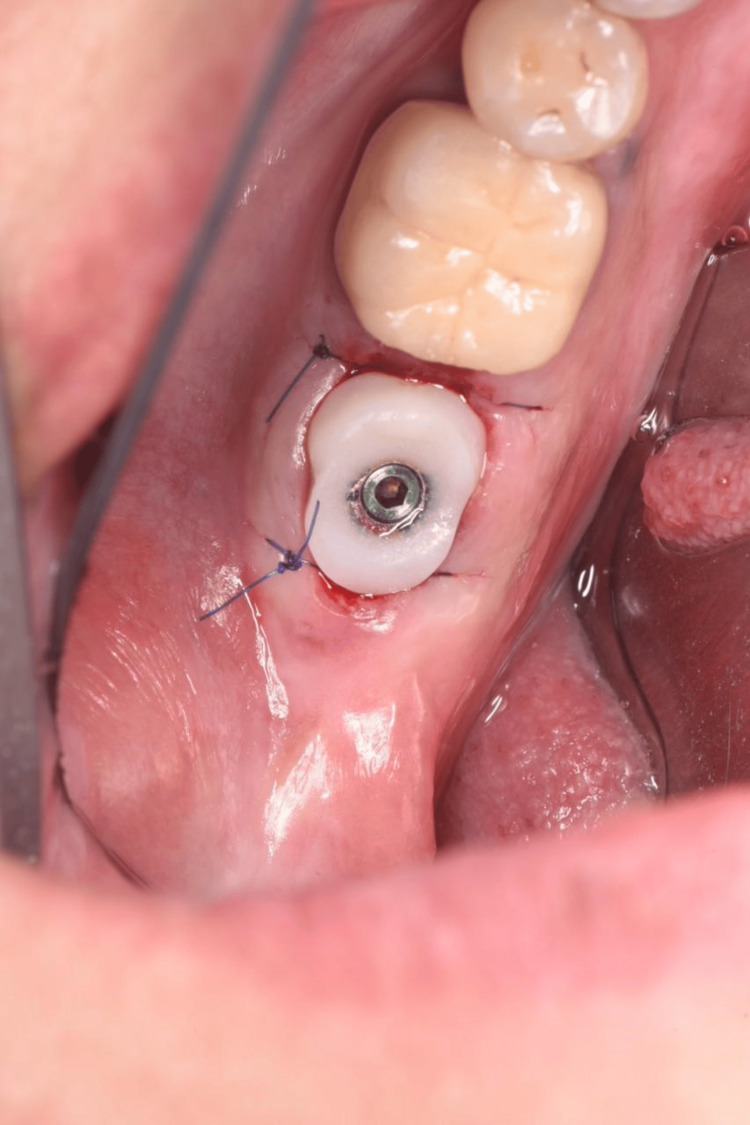
Screw-retained customized healing abutment in place.

**Figure 18:**
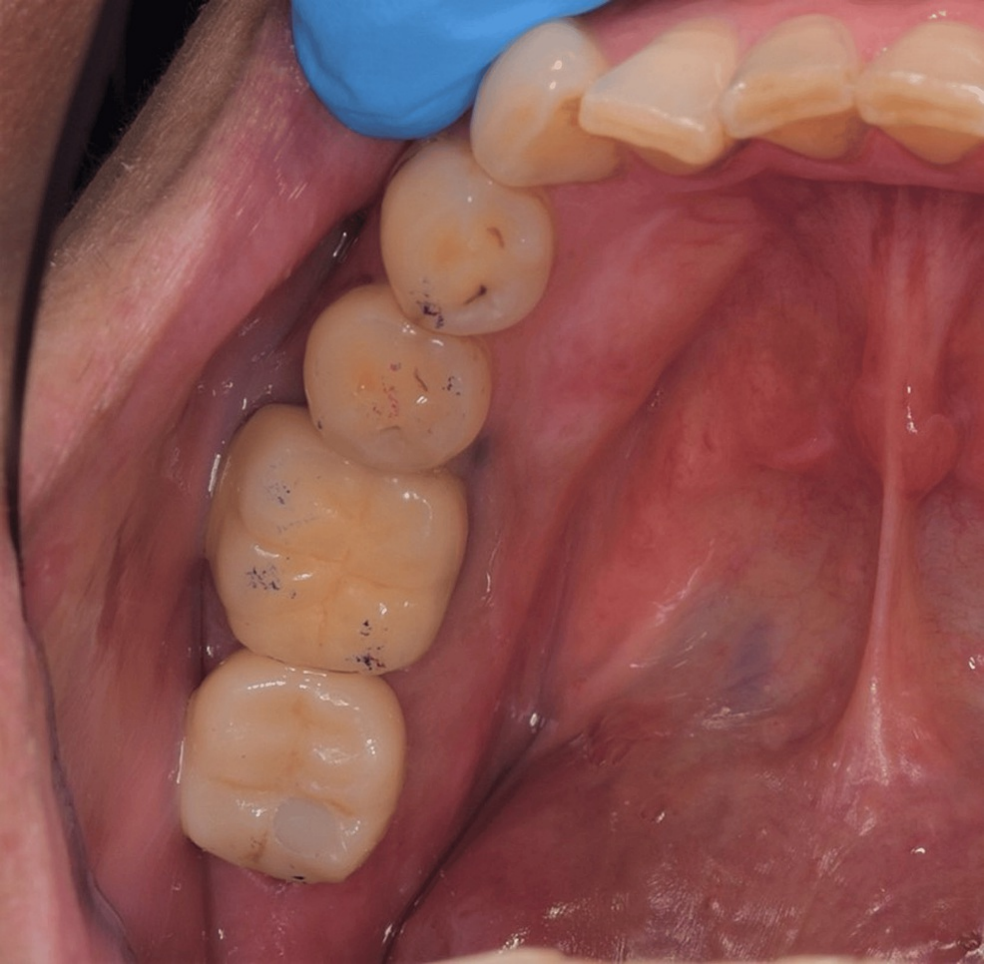
Definitive restoration (screw-retained zirconium crown).

**Figure 19:**
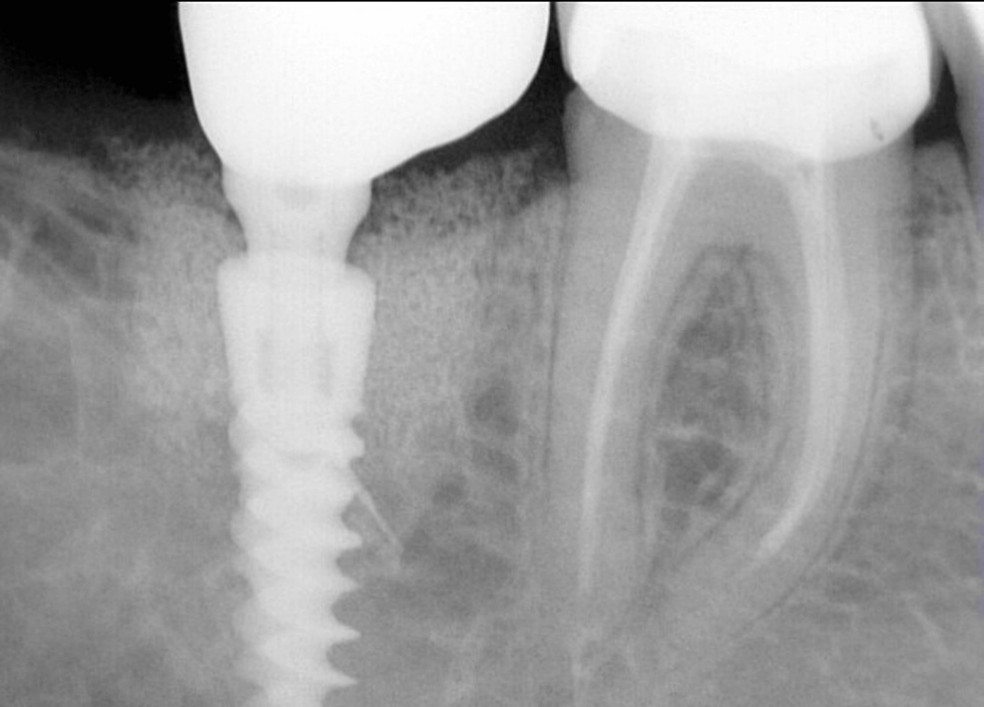
Periapical X-ray showing the implant with the definitive restoration.

**Figure 20:**
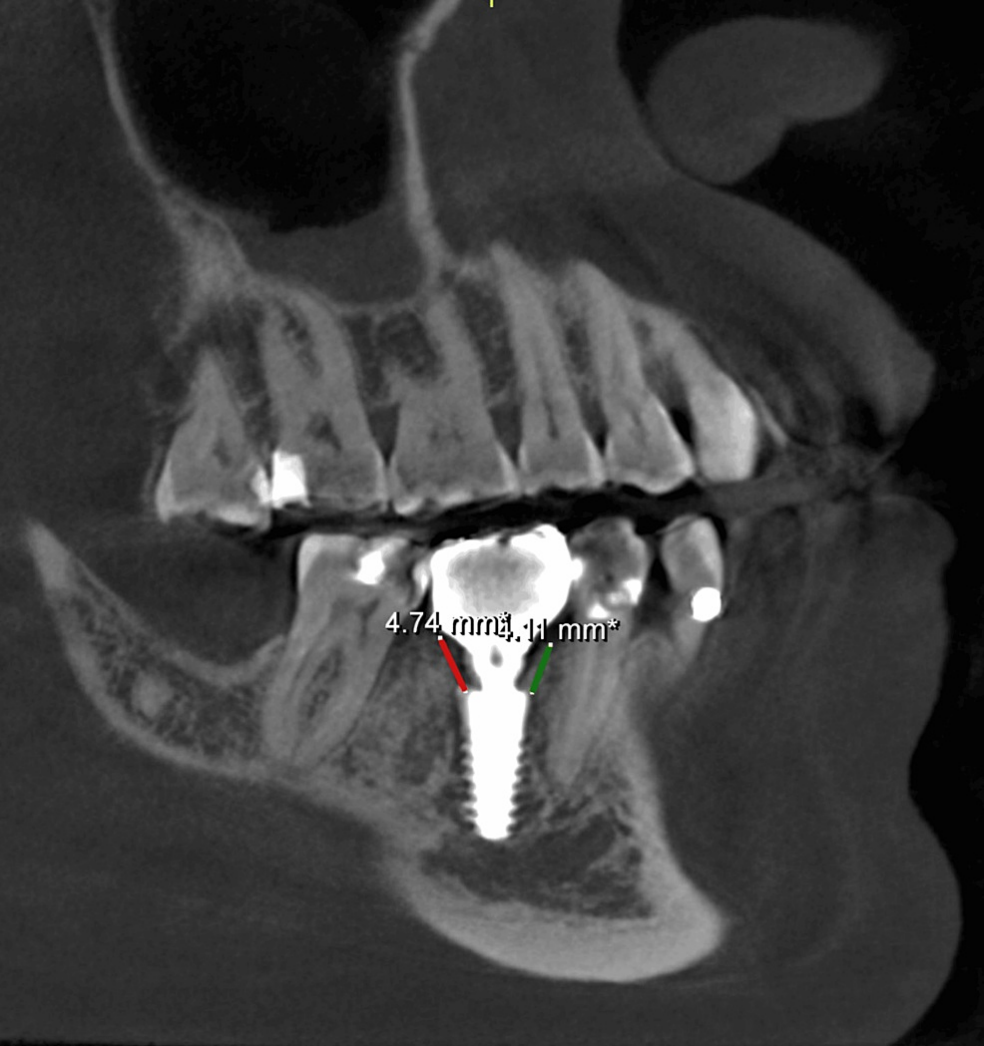
Cone beam CT scan showing the marginal bone-level measurements in mesial and distal sites.

All cases were followed up for three years after placement of the definitive restoration. The outcomes assessed in each follow-up visit were according to the newly introduced implant dentistry core outcome set and measurement (ID-COSM) [9]. The outcomes evaluated were surgical complications and peri-implant marginal tissue health status. The latter was assessed based on three domains: the presence or absence of bleeding or suppuration on probing, probing pocket depth (PPD), and marginal bone level. The marginal bone level was measured from the implant platform to the bone crest level in mesial and distal sites on the cone beam computed tomography scans. Marginal bone loss was calculated from baseline changes over time. All measurements were taken in millimeters, as illustrated in Figure 20.

Measurements were recorded at baseline (T0, immediately after definitive restoration placement), after one year (T1), after two years (T2), and three years (T3). Implant or prothesis loss was recorded over the different follow-up periods.

Statistical analysis was done using R and R Studio software [10,11]. Data organization, manipulation, and summarization were done using the “tidyverse” R package. Continuous data were summarized into mean and standard deviation. Graphs were constructed using the “ggpubr” R package, and the mean values of marginal bone loss were compared using repeated measures ANOVA from the “rstatix” R package.

## Discussion

Regarding the presence or absence of bleeding or suppuration on probing, all 11 implants showed no bleeding or suppuration at different time points, with no loss of implant or prosthesis reported. Results of the probing pocket depth and marginal bone levels are shown in Table 1.

**Table 1:** Peri-implant marginal tissue health results over a three-year follow-up period. No significant differences were obtained using the repeated measure ANOVA test. SD: standard deviation, mm: millimeter.

Probing pocket depth (mm)	Mean (SD)	Minimum	Maximum
T0 (baseline)	3.32 (0.25)	3 mm	3.5 mm
T1 (after one year)	3.23 (0.34)	2.5 mm	3.5 mm
T2 (after two years)	3.14 (0.32)	2.5 mm	3.5 mm
T3 (after three years)	3.32 (0.34)	2.5 mm	3.5 mm
Marginal bone level (mm)	Mean (SD)	Minimum	Maximum
T0 (baseline)	Mesial: 3.01 (1.02)	Mesial: 1.79 mm	Mesial: 4.65 mm
	Distal: 2.81 (0.95)	Distal: 1.74 mm	Distal: 4.95 mm
T1 (after one year)	Mesial: 3.11 (0.94)	Mesial: 1.79 mm	Mesial: 4.4 mm
	Distal: 2.67 (0.97)	Distal: 1.48 mm	Distal: 4.83 mm
T2 (after two years)	Mesial: 2.93 (0.91)	Mesial: 1.79 mm	Mesial:4.4 mm
	Distal: 2.68 (0.94)	Distal: 1.28 mm	Distal: 4.8 mm
T3 (after three years)	Mesial: 3.07 (0.90)	Mesial: 1.9 mm	Mesial: 4.5 mm
	Distal: 2.92 (1.04)	Distal: 1.72 mm	Distal: 4.85 mm
Marginal bone loss -loss/+gain (mm)	Mean (SD)	Minimum	Maximum
T1–T0	Mesial: 0.10 (0.42)	Mesial: -0.25 mm	Mesial: 1.26 mm
	Distal: -0.14 (0.67)	Distal: -1.48 mm	Distal: 0.8 mm
T2–T0	Mesial: -0.07 (0.37)	Mesial: -0.63 mm	Mesial: 0.76 mm
	Distal: -0.12 (0.70)	Distal: -1.68 mm	Distal: 0.63 mm
T3–T0	Mesial: 0.07 (0.55)	Mesial: -0.55 mm	Mesial: 1.48 mm
	Distal: 0.11 (0.79)	Distal: -1.2 mm	Distal: 1.91 mm

**Figure 21:**
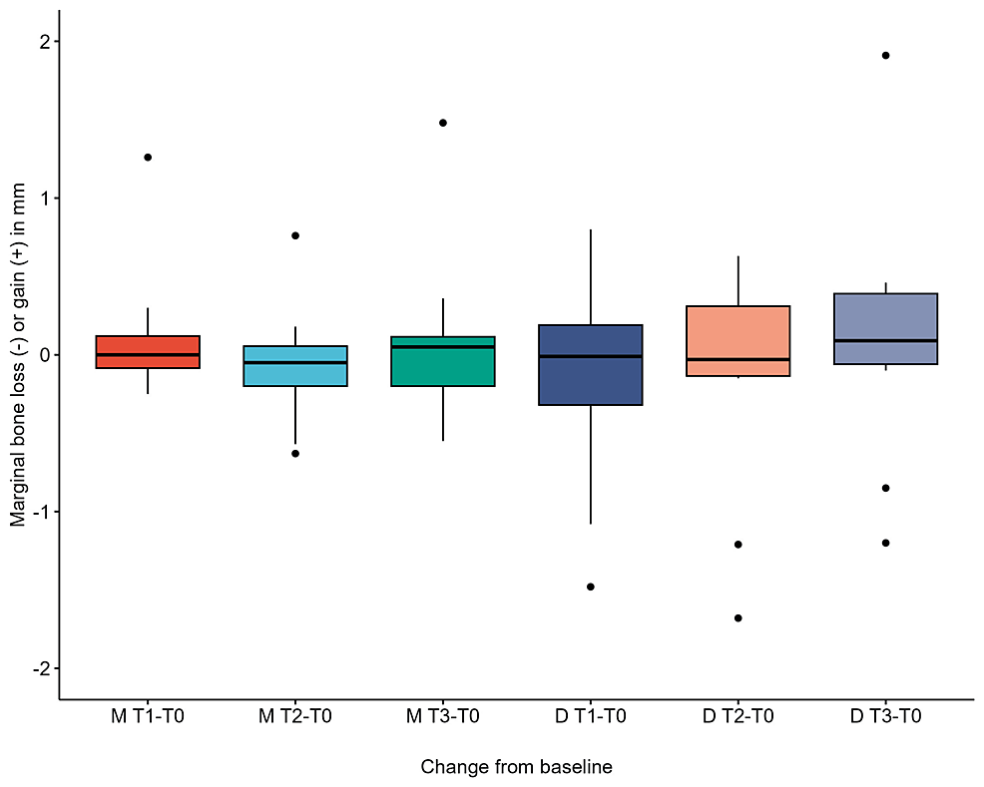
Marginal bone loss in mesial and distal sites boxplot. M: mesial, D: distal, T0: baseline, T1: after one year, T2: after two years, T3: after three years.

Marginal bone loss change from the baseline boxplot is shown in Figure 21.

This study presents 11 immediate implants placed in the posterior region using the DZT technique to replace non-restorable molars and premolars after atraumatic extraction. Using the DZT grafting technique can limit changes in the buccal contour of the extraction site and potentially improve the soft tissue thickness around the implant-abutment interface with a better emergence profile [5]. Few studies have implemented the DZT technique in immediate implants, with existing research limited to investigations within the aesthetic zone [5-8]. No studies have applied the DZT technique for immediate implant placement in posterior extraction sockets.

Various factors influence the success of immediate implant placement in posterior extraction sockets. Atraumatic tooth extraction, flapless technique, and primary implant stability are crucial for its success [3,12]. The piezotome-based atraumatic extraction technique is preferred for immediate implant placement, ensuring minimal damage to the surrounding soft and hard tissues. Research reveals that this technique takes longer than conventional methods, resulting in better tissue preservation and lesser marginal bone loss after implant placement [13-16]. Using a flapless technique in tooth extraction and implant placement can reduce postoperative tissue loss, shorten operative time, speed up healing, lower the risk of complications, and improve patient comfort [1]. Root-form implants with aggressive threads are essential for immediate implant placement to achieve stability by ensuring more primary stability and a larger implant surface area in engaging the socket walls [3].

The type of bone graft used was a bovine xenograft with 0.5 to 1 mm particle size, which coincides with the kind used by Chu and colleagues [5]. In the DZT technique, grafting the jumping gap to the free gingival margin can help reduce the collapse of the ridge in the bucco-palatal dimension. This technique can increase the thickness of soft tissues around implants, preventing discoloration and the need for invasive grafting procedures [3]. A recent systematic review concluded that using bone-substitute materials in immediate implant placement resulted in less horizontal buccal bone resorption and better peri-implant soft tissue esthetics than no bone-substitute placement [17].

A screw-retained custom healing abutment offered a prosthetic seal to contain and protect the graft during the immediate implant placement's healing phase and decrease the gingival tissue's collapse and recession [18]. Using screw-retained abutments is more beneficial than cement-retained ones to minimize movement and microleakage with only one interface [3]. The implant loading protocol was delayed after four to six months, which has a better survival rate than the immediate loading [1].

The outcomes assessed in this study followed the recently published core outcome set in implant dentistry (ID-COSM) [9], reflecting the benefits and harms of implant interventions. During the three-year follow-up period, no surgical complications or implant/prosthesis loss were observed, reflecting a high success rate of immediate implant placement in the posterior region. This agrees with a recent systematic review showing a 96.6% survival rate and 93.3% success rate after one year of follow-up [1]. Peri-implant marginal tissue health status showed promising results over the three-year follow-up period. No bleeding/suppuration was observed, and PPD ranged from 2.5 mm to 3.5 mm. These results could be due to packing the graft material in the jumping gap until the free gingival margin with a customized healing abutment that acted as a prosthetic seal, benefiting both the graft and the gingival tissues [3].

The marginal bone loss showed minimal mean values ranging from 0.11 mm gain to a 0.14 mm loss during the three-year follow-up, and some cases showed bone loss of up to 1.68 mm and gain of up to 1.91 mm. The mean values of marginal bone loss in our study differed from those reported by Ragucci and colleagues, with a mean value of 1.29 mm. However, our study only assessed 11 immediately placed implants [1]. Ragucci and colleagues also demonstrated that grafting the jumping gap in immediate implant placement favored more success and survival of implants than no grafting, which could reflect on our results [1]. Another case series involving 15 immediate implants placed in molar extraction sockets has shown minimal marginal bone loss of 0.17 mm over one year, which aligns with our findings. However, the previous study reported the failure of four implants that had to be removed before the first year, in contrast to our findings of no implant loss or any complications over three years [19].

## Conclusions

The DZT technique implemented with immediate implants in posterior extraction sockets showed promising peri-implant marginal tissue health results with no observed complications or implant loss over three years. This technique is easy to apply and depends on grafting the jumping gap to the free gingival margin using a customized healing abutment as a prosthetic sealing device. Further research with larger sample sizes should compare the DZT technique in posterior extraction sockets to alternative approaches.
